# THE ROLE OF PERCEIVED SOCIAL SUPPORT AND PRUDENT DIET INTAKE ON ALLOSTATIC LOAD AMONG OLDER ADULTS

**DOI:** 10.1093/geroni/igz038.322

**Published:** 2019-11-08

**Authors:** Danielle D'Amico, Vivian Huang, Geneva Millett, Alexandra J Fiocco

**Affiliations:** Ryerson University, Toronto, Ontario, Canada

## Abstract

Allostatic load (AL), an index of multisystem physiological dysregulation due to chronic stress, has been identified as a predictor of poor health outcomes in late life. Research suggests that perceived social support (PSS) improves health outcomes by buffering the negative effects of stress on wellbeing and increasing health promoting behaviours including consumption of a healthy prudent diet (i.e., fruits, vegetables, lean meats, nuts, and seeds). Research to date has independently demonstrated that higher PSS and prudent diet intake have an effect on AL. A paucity of research, however, has examined how dietary consumption and PSS interact to effect AL in older adults. The objective of this study was to examine the interaction between PSS and prudent diet pattern on AL in 164 non-demented, community-dwelling older adults (Mage= 68.5(.52), 64% female). PSS and diet intake were measured using the Perceived Social Support Scale and the EPIC-Norfolk Food Frequency Questionnaire, respectively. AL was composed of 16 biomarkers stemming from neuroendocrine, metabolic, inflammatory, and cardiovascular systems, stratified by sex. Controlling for age and usual daily energy intake, higher prudent diet consumption (B=-2.04, p=.001), but not PSS, was associated with lower AL. Moderation analysis revealed that higher prudent diet intake was associated with lower AL only for those with low PSS (B=-.83, p=.0006) and mean level of PSS (B=-.43, p=.02). These findings suggest that chronic biological stress may be mitigated by consuming a healthy diet specifically for older adults with lower social support and may further inform intervention strategies to promote healthy aging.

